# Rapid Screening of Nitrogen Use Efficiency in Perennial Ryegrass (*Lolium perenne* L.) Using Automated Image-Based Phenotyping

**DOI:** 10.3389/fpls.2020.565361

**Published:** 2020-08-27

**Authors:** Junping Wang, Adam M. Dimech, German Spangenberg, Kevin Smith, Pieter Badenhorst

**Affiliations:** ^1^Agriculture Victoria Research, Hamilton, VIC, Australia; ^2^AgriBio Centre for AgriBioscience, Agriculture Victoria Research, Bundoora, VIC, Australia; ^3^School of Applied Systems Biology, La Trobe University, Bundoora, VIC, Australia; ^4^The Faculty of Veterinary and Agricultural Sciences, The University of Melbourne, Parkville, VIC, Australia

**Keywords:** perennial ryegrass, biomass, imaging, nitrogen use efficiency, phenomics

## Abstract

Perennial ryegrass (*Lolium perenne* L.) is a dominant species in temperate Australian pastures. Currently, nitrogenous fertilizers are used to support herbage production for pasture and fodder. Increasing the nitrogen use efficiency (NUE) of pasture grasses could decrease the amount of fertilizer application and reduce nitrogen (N) leaching into the environment. NUE, defined as units of dry matter production per unit of supplied nitrogen, is a complex trait in which genomic selection may provide a promising strategy in breeding. Our objective was to develop a rapid, high-throughput screening method to enable genomic selection for y -60NUE in perennial ryegrass. NUE of 76 genotypes of perennial ryegrass from a breeding population were screened in a greenhouse using an automated image-based phenomics platform under low (0.5 mM) and moderate (5 mM) N levels over 3 consecutive harvests. Significant (*p* < 0.05) genotype, treatment, and genotype by treatment interactions for dry matter yield and NUE were observed. NUE under low and moderate N treatments was significantly correlated. Of the seven plant architecture features directly extracted from image analysis and four secondarily derived measures, mean projected plant area (MPPA) from the two side view images had the highest correlation with dry matter yield (r = 0.94). Automated digital image-based phenotyping enables temporal plant growth responses to N to be measured efficiently and non-destructively. The method developed in this study would be suitable for screening large populations of perennial ryegrass growth in response to N for genomic selection purposes.

## Introduction

Global population growth demands a continuing increase in the efficiency of agricultural production. The application of nitrogenous fertilizers to crops and pastures can improve productivity, but also adds to the cost of production whilst contributing to nutrient runoff and the emission of nitrous oxide, a greenhouse gas, through denitrification. Global total nitrogen (N) consumption and average application rate on cropland increased more than eight times between 1961 and 2013 (Lu and Tian, 2017). The N nutrition of dairy pasture systems in southern Australia relies almost completely on N fertilizer today (Rawnsley et al., 2019). Therefore, the challenge remains of how to achieve high production with less cost and lower environmental impact. Several strategies have been identified that could reduce the demand for N fertilizer in agriculture. For plant breeders, the selection and breeding of plant cultivars that capture N and convert it to protein more efficiently would offer considerable benefit. Raun and Johnson (1999) demonstrated that a 1% increase in nitrogen use efficiency (NUE) in cereal production worldwide would result in US $235 million in savings from reduced annual applications of N fertilizer in addition to the environmental and social benefits.

NUE is known to be a complex quantitative trait and many genes are involved including for nitrogen signalling, uptake, transport/remobilization, and transcription factors (Han et al., 2015; Liu et al., 2015; Li et al., 2017). For a quantitative trait, genomic selection (GS) provides a promising strategy in breeding (Hayes et al., 2013). However, GS depends on the availability of an accurate and high-throughput phenotyping approach to be successful, which has historically been a challenging bottleneck. New technologies in image-based phenotyping are now allowing researchers to overcome these experimental limitations.

Perennial ryegrass is a dominant species in temperate pastures. N fertilizer greatly impacts perennial ryegrass growth, development and chemical composition, which is related to herbage quality for grazing livestock. A widely adopted application rate recommendation was 20–60 Urea-N kg ha^-1^ after grazing (Rawnsley et al., 2019). Sometimes the rates are greater in more intensive systems (Gislum et al., 2003; Gourley et al., 2017). Differences in NUE among perennial ryegrass cultivars after defoliation were reported using hydroponics (Wilkins et al., 1997) and field trials under variable N application rates (Wilkins et al., 2000). The variation among cultivars indicated selection and breeding for improving NUE *via* GS would be possible. However, these researchers were working at the cultivar level and only a limited number were tested whilst the methodologies were not high throughput. Development of GS for improving NUE in perennial ryegrass requires rapid and high-throughput effective screening methods.

Plant phenotyping in controlled environments can be particularly useful in studying different genotypes in response to specific conditions and eliminating variation caused by other factors. For the past decade, there has been increased research into applying image-based high-throughput phenotyping in controlled environments for different crops and traits (Das Choudhury et al., 2019). Recently, an automated image-based system was used to screen NUE for wheat varieties at the vegetative phase (Nguyen et al., 2019). To date, there have been limited reports of using such systems for forage grasses, where unique challenges exist due to differences in plant architecture such as dense tillers, occlusion of leaves, and regrowth after repetitive defoliation. Traditionally, pasture grass response to N supply is measured as a biomass yield by destructively harvesting the foliage. Non-destructive measurements such as tiller count and leaf area estimates have also been applied for perennial ryegrass. However, these measurements still involve manual handling of plants and may cause inadvertent damage. Automated digital image-based analysis offers a non-destructive, high throughput, and less invasive option.

The objectives of this study were to develop an image-based method for rapid NUE screening for perennial ryegrass and to explore the genetic variation of NUE within an advanced breeding population under low and moderate N levels.

## Materials and Methods

### Plant Materials

Seventy-six perennial ryegrass genotypes from an advanced breeding population were used in this study. Plants were initially grown in a greenhouse in plastic pots (120 mm diameter) with commercial potting mix (BioGrow Australia Pty. Ltd., Mount Gambier, South Australia, Australia) at Agriculture Victoria, Hamilton Centre, Hamilton, Victoria, Australia. Each plant was propagated into 8 clonal ramets of 5 tillers each into forestry tubes (50 × 50 × 120 mm) three weeks before setting up the experiment. Plants were grown in the same greenhouse with temperature set to 22°C under ambient light conditions.

### Imaging Greenhouse and Experiment Design

**Figure 1:**
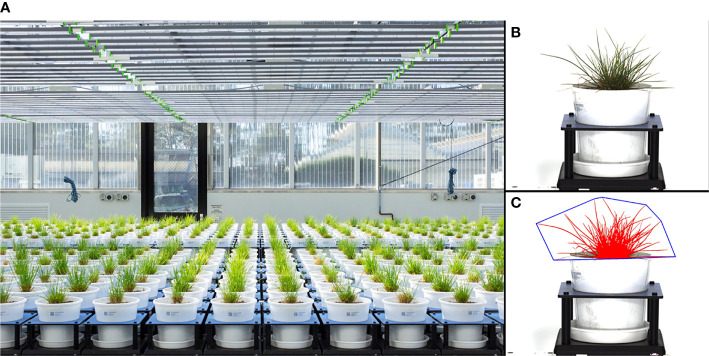
Imaging greenhouse **(A)**, a raw side-view image of a perennial ryegrass plant **(B)**, and the processed image object from the raw image **(C)**. The diameter of the pots is 200 mm.

The phenotyping experiment was conducted at Plant Phenomics Victoria, Bundoora, Victoria, Australia, which is equipped with two greenhouses containing a LemnaTec 3D Scanalyzer phenomics platform (LemnaTec, GmbH, Aachen, Germany; **Figure 1**).

A randomized complete block design was used with two N levels and four replicates. A total of 608 pots were laid out into 16 rows with 38 pots per row. Each replicate consisted of four rows, i.e. two rows for each of the low N and moderate N treatments. The 76 genotypes were randomly allocated within each of the treatment rows. Two levels of N treatments were alternated between the rows.

### Treatment and Growth Conditions

Plants were cut to 5 cm height at the commencement of the experiment and transplanted into white 200 mm diameter pots (catalogue P200E04, Garden City Planters Pty. Ltd., Dandenong South, Victoria, Australia). Pots were filled with potting mix containing coir peat, composted pine bark, composted sawdust, SaturAid^®^ (soil-wetting agent), lime, and gypsum (Australian Growing Solutions Pty. Ltd., Tyabb, Victoria, Australia). No nutrient components were added to the potting mix. Pots were placed into carriers which were tagged with radio frequency identification (RFID) chips for tracking and moved in the greenhouse on the LemnaTec Scanalyzer 3D *via* a series of conveyors to watering and imaging stations. Watering was controlled programmatically and checked daily *via* weighing scales, which allowed estimates of evapotranspiration to be calculated for each individual pot (Ge et al., 2016). Plants were grown in the greenhouse at 22°C for 16 h (day) and 15°C for 8 h (night). Natural sunlight was supplemented with full-spectrum light-emitting diode (LED) growth lights (Samsung, Seoul, South Korea) with an approximate photosynthetic photon flux of 600 µmol m^-2^ s^-1^ at canopy height when it fell below 500 µmol m^-2^ s^-1^.

Half-strength Hoagland solution (Hoagland and Arnon, 1950) (100 ml) with modified N levels of 0.5 mM or 5 mM (Jiang et al., 2016) was applied to each pot twice weekly. KNO_3_ and Ca(NO_3_)_2_ were used as nitrate sources in the solution. Potassium and calcium levels were equalized across treatments by adding KCl and CaCl_2_ into the lesser N treatment solution (Jiang et al., 2016).

### Image Acquisition and Analysis

Imaging was conducted twice a week *via* a series of visible-spectrum (red-green-blue, RGB) cameras (Prosilica GT, Allied Vision Technologies GmbH, Stadtroda, Germany) fitted with a 50 mm focal lens (T* 250 ZF, Carl Zeiss AG, Oberkochen, Germany) and located within an imaging cabinet. RGB images of each plant were taken from above (top view) and from the side at two angles (0° and 90°). Plant images were analyzed using a customized grid programmed within LemnaGrid software before the plant architecture features (**Table 1**) were extracted. The imagery data were treated as repeated measures.

**Table 1:** Features extracted from digital RGB images of perennial ryegrass plants.

Trait	Unit	Description
Area	pixel	Projected plant area from top or side view images
Calliper Length	pixel	The longest dimension of the projected canopy
Compactness		A ratio of projected plant area to convex hull area
Convex Hull Area	pixel	Projected minimum area contained the object
Convex Hull Circumference	pixel	Circumference of the projected convex hull
Width	pixel	Projected maximum length horizontally
Height	pixel	Projected maximum length vertically

The following derived volume estimations, which have been reported correlated to biomass in maize (Klukas et al., 2014), field pea (Nguyen et al., 2018) and wheat (Nguyen et al., 2019) were calculated.

$$V=A_{s.0^{\circ }}+A_{s.90^{\circ }}+A_{t}$$

$$V_{lemnatec}=\sqrt{A_{s.0^{\circ }}\times A_{s.90^{\circ }}\times A_{t}}$$

$$V_{\text{keygene}}=A_{s.0^{\circ }}+A_{s.90^{\circ }}+\log\left(\frac{{\text{A}}_{\text{t}}}{3}\right)$$

Where *A_s.0°_* is the projected area from the 0° side view image, *A_s.90°_* is the projected area from the 90° side view image, and *A_t_* is the projected area from the top view image.

### Destructive Harvests and Nitrogen Use Efficiency

The total duration of the experiment was 3 months. Three manual cuts of individual plants were conducted at approximately 4-week intervals and at 5 cm height from the potting mix. Chronologically, the corresponding three regrowth periods were defined as acclimation phase (AP), experimental phase-1 (EP-1), and experimental phase-2 (EP-2). The fresh biomass (FM) of plant material was measured and samples were then dried at 60°C for 48 h and dry biomass (DM) was weighed. The AP allowed plants to establish after transplanting and acclimate to the experimental conditions, whilst also permitting the consumption of any residual N from the root surface and metabolization of excess N stored in the roots and crown to be completed so that a proper assessment of the N treatments in EP-1 and EP-2 could be made.

NUE was calculated in both EP-1 and EP-2 as follows:

$$NUE=\frac{DM(g)}{N_{S}(g)}$$

where DM is the dry biomass, and N_s_ is the supplied N.

Seven applications of the nutrient solution were applied to each pot during each of the experimental phases, which is equivalent to 4.9 mg N per pot in the low N treatment and 49 mg N in the moderate N treatment. The application rate of 49 mg N per pot is equivalent to approximately 20 kg/ha.

### Plant Growth Analysis

Absolute plant growth rate (AGR) on DM (g d^-1^) in EP-1 and EP-2 were calculated as average accumulated DM per day during their respective growth periods. AGR on mean projected plant area (MPPA, pixel d^-1^) from the two side view images was calculated as follows:

$$AGR=\frac{MPPA_{2}-MPPA_{1}}{t_{2}-t_{1}}$$

and relative growth rate (RGR) on MPPA was calculated as follows:

$$RGR=\frac{Ln(MPPA_{2})-Ln(MPPA_{1})}{t_{2}-t_{1}}$$

where *t_2_* and *t_1_* were the respective finish and start time points of a growth period, Ln(MPPA_2_) and Ln(MPPA_1_) was the natural logarithm of MPPA at the time of *t_2_* and *t_1_* respectively. The RGR results were given as per day (i.e. 0.2 d^-1^ is equivalent to 20% increase in a day) (Poiré et al., 2014).

### Statistical Analysis

Statistical analysis was conducted using GenStat v.18 (VSN International, 2015). Linear mixed model analysis was conducted using a restricted maximum likelihood (REML) approach in GenStat where N treatment level, genotype and their interaction were fitted as fixed factors and replicate as random factor. The best linear unbiased estimations (BLUEs) of DM and NUE for each genotype under low and moderate N levels were obtained from REML and plotted in Microsoft Excel. Pearson’s correlation coefficient (*r*) between features extracted from images and FM and DM were computed and plotted using the “PerformanceAnalytics” package in R statistical software (version R-3.5.1) (R Core Team, 2017).

## Results

### Perennial Ryegrass Genotype Response to Different N Supplies

**Figure 2:**
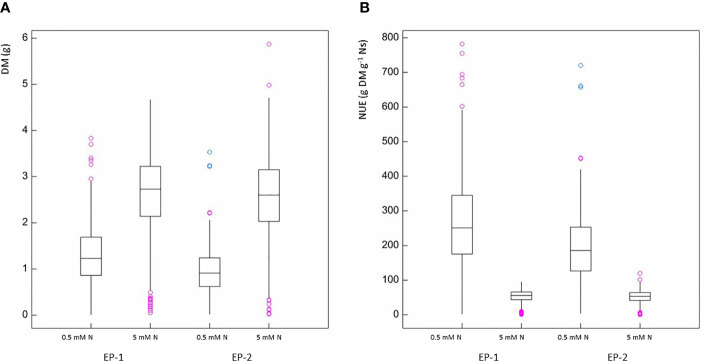
Box and whisker plots of dry biomass **(A)** and nitrogen use efficiency **(B)** in experimental phase-1 (EP-1) and experimental phase-2 (EP-2) under two levels of N treatment (0.5 mM and 5 mM N).

REML analysis showed that DM was significantly greater in plants with moderate N compared to those with low N supply (p < 0.001) in both EP-1 and EP-2 (**Figure 2A**). Different genotypes within each treatment also showed a significant difference in DM (p < 0.001) and the genotype by N treatment interaction was also significant (p < 0.001). In contrast, NUE was signicantly less in moderate N supply than that in low N supply (p < 0.001) in both EP-1 and EP-2 (**Figure 2B**). Genotype effect was significant for NUE (p < 0.001) in both EP-1 and EP-2 and genotype by N interaction was also significant in EP-1 (p < 0.001) and in EP-2 (p < 0.05).

**Figure 3:**
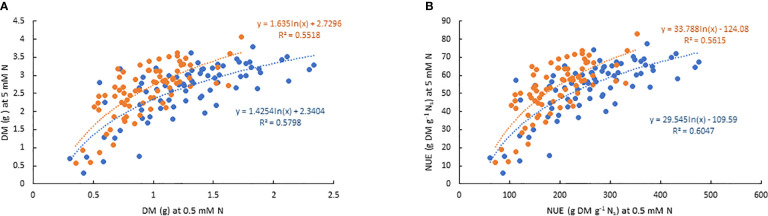
Scatter plots of best linear unbiased estimates (BLUEs) of dry biomass **(A)** and nitrogen use efficiency **(B)** of 76 genotypes under two levels of N treatment over the two experimental phases (Blue dots: EP-1; orange dots: EP-2). BLUEs were derived from the restricted maximum likelihood (REML) in GenStat where genotype, N treatment and their interaction were fitted as fixed factors and replicate was fitted as random factor for each variate in each experimental phase.

There was significant variation among genotypes for DM and NUE under both low and moderate N supplies (**Table 2**). The estimates of NUE under low N supply were in a range of 61.7–476.5 and 72.4–353.1 g DM g^-1^ Ns in EP-1 and EP-2, respectively. The population mean were significantly lower in EP-2. The estimates of NUE under moderate N supply were in a range of 6.0–77.4 and 11.8–83.0 g DM g^-1^ Ns in EP-1 and EP-2, respectively. There was no difference of population means between EP-1 and EP-2. In terms of NUE, the best genotype was over 4 times and 7 times greater than that of the worst geotype under low and moderate N supply, respectively. The NUE variance in EP-1 was two-fold of that in EP-2 under low N whilst the variance was no different between EP-1 and EP-2 under moderate N. The correlations of DM and NUE between low and moderate N treatments were significant in both EP-1 and EP-2 (**Figure 3**).

**Table 2:** Summary statistics of best linear unbiased estimations of dry biomass (DM) and nitrogen use efficiency (NUE) of 76 genotypes of a perennial ryegrass breeding population under two levels of N in two experimental phases (EP-1 and EP-2).

	N Level	DM (g)			NUE (g DM g^-1^ Ns)		
		Mean ± S.D.	Range	CV	Mean ± S.D.	Range	CV
EP-1	0.5 mM	1.29 ± 0.446	0.30–2.34	0.35	263.3 ± 91.18	61.7–476.5	0.35
	5 mM	2.60 ± 0.755	0.30–3.80	0.29	52.99 ± 15.34	6.0–77.4	0.29
EP-2	0.5 mM	0.95 ± 0.295	0.36–1.73	0.31	194.5 ± 60.22	72.4–353.1	0.31
	5 mM	2.57 ± 0.720	0.58–4.07	0.28	52.31 ± 14.74	11.8–83.0	0.28

S.D., standard deviation; CV, coefficient variation.

### Relationships Between Features Extracted From Images and Biomass of Perennial Ryegrass Plants

**Figure 4:**
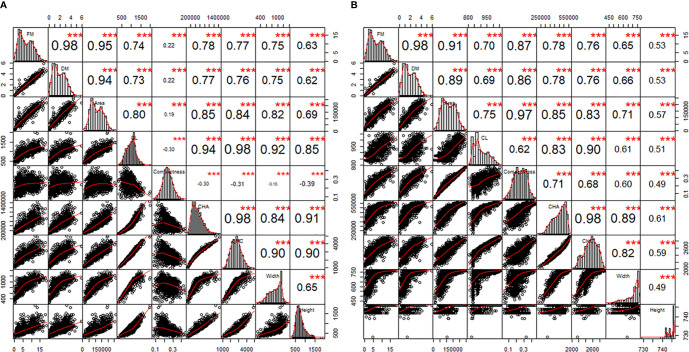
The correlations between features from 0°-side view **(A)** and top view **(B)** images and fresh and dry biomass over the two experimental phases. FM, fresh biomass; DM, dry biomass; CL, calliper length; CHA, convex hull area; CHC, convex hull circumference. In each panel, the diagonals are the histograms of individual traits. The windows above and below the diagonals are Pearson’s correlation coefficients (r) and bivariate scatter plots with non-parametric regression smooth lines, respectively. The asterisks are the statistically significant levels (***p < 0.001). Sample number = 1164.

**Figure 5:**
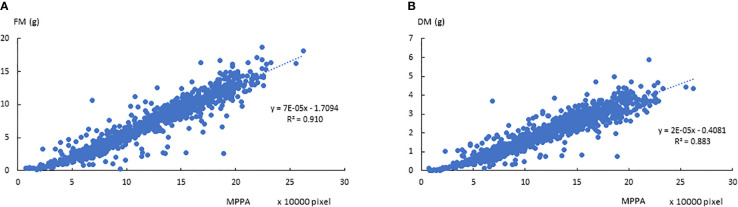
Relationships between the mean projected plant area (MPPA) from 0° and 90°-side view images and fresh biomass (FM, A) and dry biomass (DM, B) over the two experimental phases.

The correlations between FM, DM, and the 7 plant architecture features (**Table 1**) from side view and top view images are shown in **Figures 4A, B**, respectively. All traits were significantly correlated with varied correlation coefficients. Out of the 7 features, the projected plant area of the side view image demonstrated the highest correlation with FM and DM. The mean projected plant area (MPPA) from the two side view images slightly improved the correlation with FM and DM than a single image alone (**Figure 5**). The derived *V*, *V_LemnaTec_* and *V_keygene_* were also highly correlated with FM and DM with correlation coefficient of 0.947, 0.946, 0.954 and 0.933, 0.928 and 0.940, respectively. *V_keygene_* was as good as the MPPA. The plant architecture features showed slightly higher correlations with FM than with DM.

### Temporal Dynamics of MPPA

**Figure 6:**
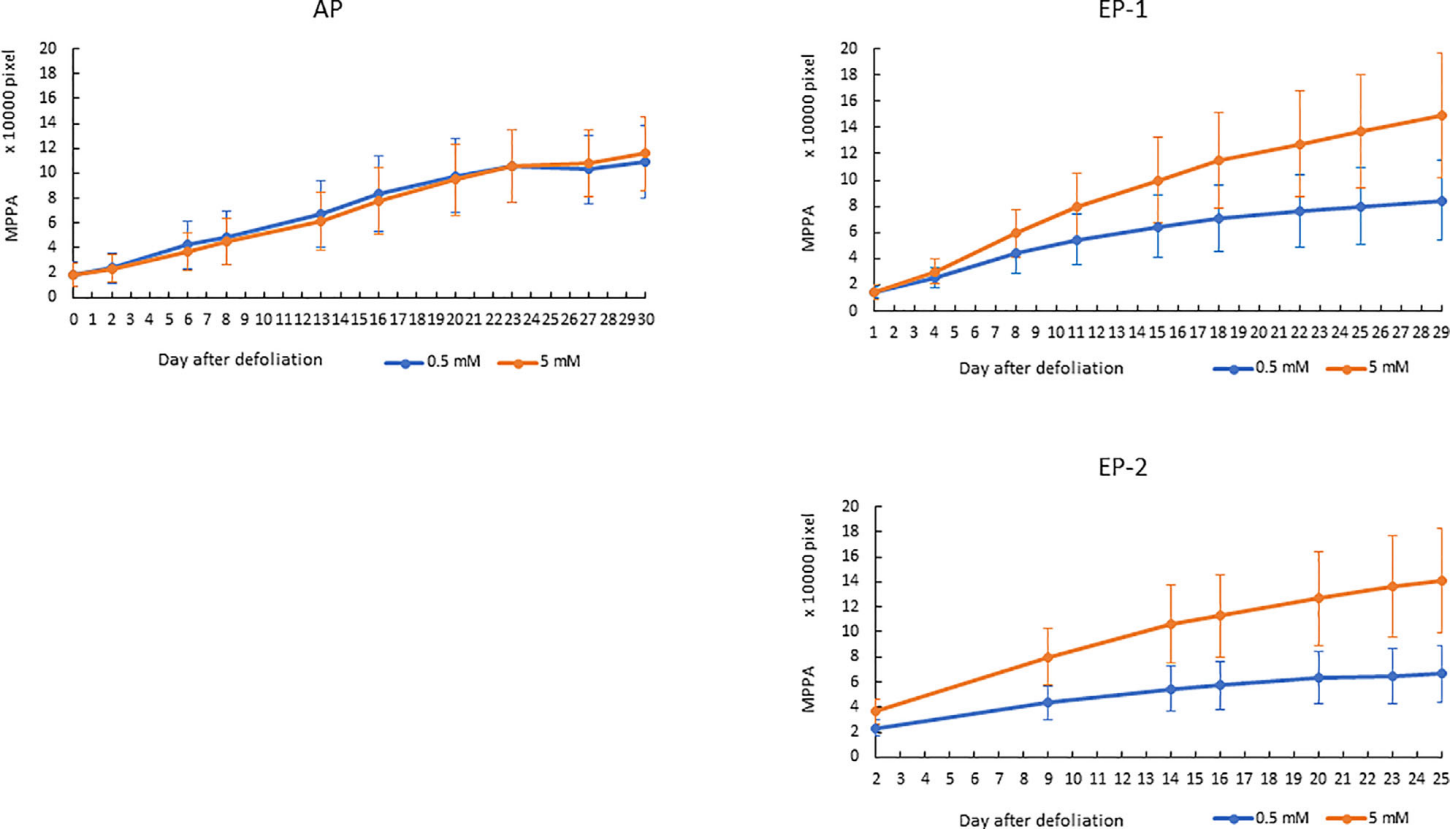
Accumulation of mean projected plant area (MPPA) from 0° and 90° -side view images in acclimation phase (AP) and experimental phase-1 (EP-1) and phase-2 (EP-2) after defoliation. The error bar is the standard deviation of the mean for the group.

**Figure 7:**
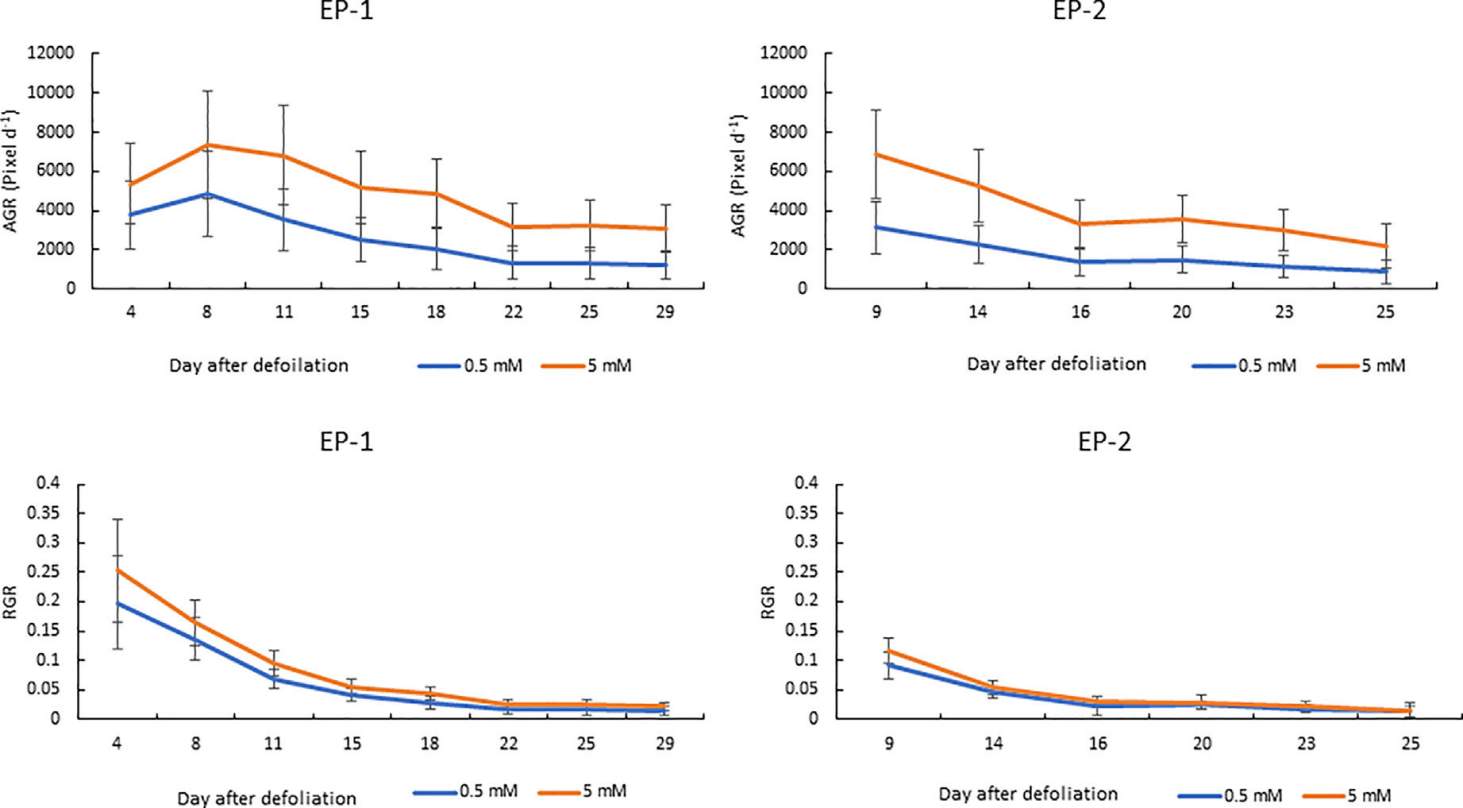
Absolute and relative growth rate (AGR and RGR) over time on the mean projected plant area (MPPA) from the two side view images under two levels of N treatment (0.5 and 5 mM) during experimental phase-1 (EP-1) and experimental phase-2 (EP-2).

Since the MPPA from the two side view images showed the highest correlation with FM and DM, the temporal dynamics of MPPA after defoliation were investigated under two N treatments for AP, EP-1 and EP-2 (**Figure 6**). In AP, MPPA accumulation showed a linear trend over time and there was no significant difference between the two nitrogen treatment levels. In EP-1 and EP-2, MPPA accumulation under the 5 mM N treatment was significantly faster than those under 0.5 mM N treatment. Under the low N treatment, MPPA continued to increase within 4 weeks of defoliation, however, the accumulation of MPPA became much slower after 3 weeks and the limited N restricted plant growth. The absolute growth rate (AGR) on MPPA increased in the first week after defoliation and then decreased thereafter under both low and moderate N (**Figure 7**). The relative growth rate (RGR) declined sharply within the 3 weeks after defoliation then decreased gradually thereafter.

**Figure 8:**
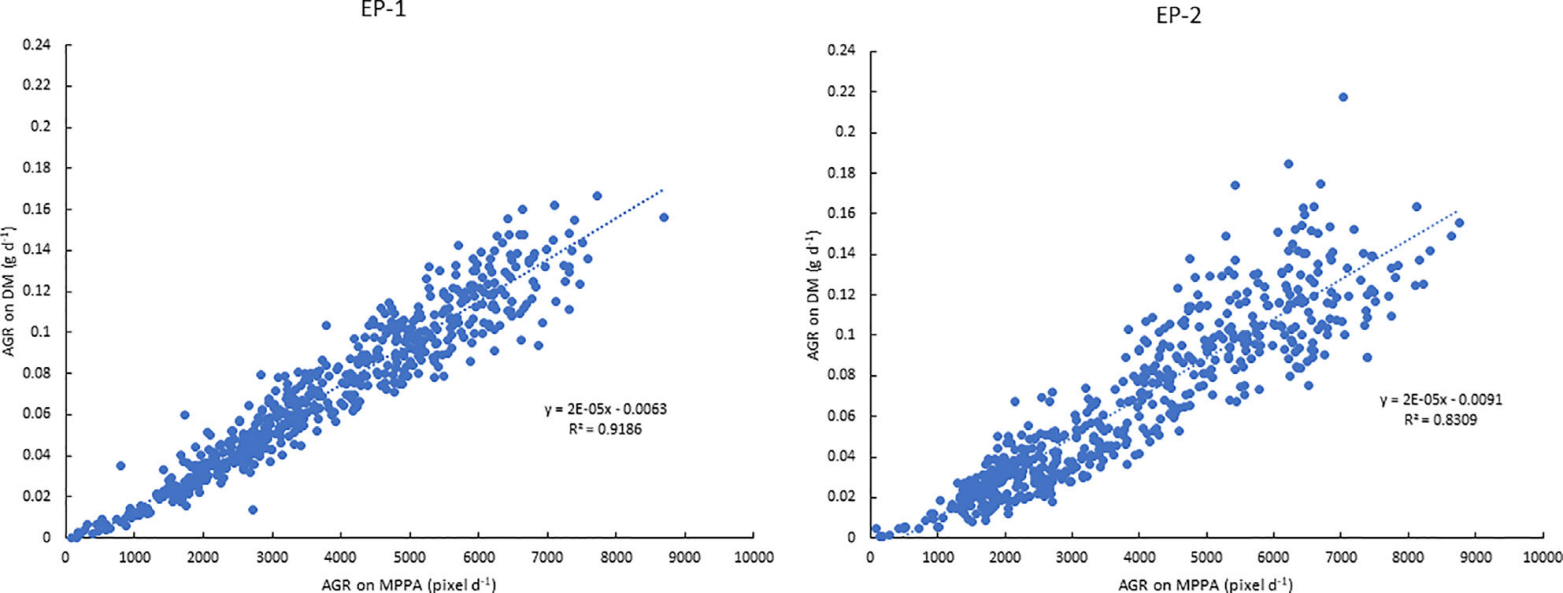
Relationships between absolute growth rate (AGR) on the mean projected plant area (MPPA) from the two side view images and on dry biomass (DM) during experimental phase-1 (EP-1) and experimental phase-2 (EP-2).

The AGR on the DM basis was 0.046 and 0.092 g d^-1^ under 0.5 and 5 mM N in the EP-1 and 0.035 and 0.094 g d^-1^ in the EP-2. AGR on the MPPA basis was 2555 and 4858 pixel d^-1^ under 0.5 and 5 mM N in EP-1 and 2310 and 5168 pixel d^-1^ in EP-2. Strong correlations between AGR on DM and MPPA were observed in EP-1 and EP-2 (**Figure 8**).

## Discussion

### Automated Image-Based Phenotyping in Controlled Environments

In this paper we describe the development of an automated image-based phenotyping methodology to screen NUE in a breeding population of perennial ryegrass.

Our results demonstrated that a LemnaTec Scanalyzer 3D image-based greenhouse phenomics platform can be used to extract trait features from images as a reliable proxy for numerous traits of interest in perennial ryegrass, including NUE. The aim of this experiment was to quantify seven plant architecture features and four derived measurements, which has been amply demonstrated. In our study, the MPPA and *V_keygene_* showed the highest correlations with FM (*r* = 0.95) and DM (*r* = 0.94). We also observed a strong correlation between digital volume and FM, as has been shown by others in *Brachypodium* (Poiré et al., 2014), sorghum (Neilson et al., 2015), field pea early stage of growth (Nguyen et al., 2018), and wheat vegetative phase (Nguyen et al., 2019). In perennial ryegrass, incorporating the digital area from the top view image did not improve the correlation with biomass yield ***via*** our MPPA metric. This may be because the perennial ryegrass canopy structure appears denser and more symmetrical when compared to wheat or sorghum. The correlation between MPPA and FM was found to be slightly greater than that between MPPA and DM. Greater correlation between image based data with FM than with DM was also found in maize (Klukas et al., 2014). This may be because the image features were based on living plants and variation in moisture content in plants were not captured in the RGB imagery. The correlation with DM may be further improved by hyperspectral images by which water content can be estimated (Ge et al., 2016). Furthermore, the features extracted were based on 2D images; 3D reconstruction of plant may provide a more accurate estimation (Gibbs et al., 2017).

Our results have demonstrated that we can use image-based phenomics to quantify biomass accumulation and growth in greenhouse-grown perennial ryegrass without the need for destructive harvesting. The savings that this brings are considerable; less labor is required to undertake research of this nature and results can be obtained faster, effectively speeding-up breeding programs.

Breeding for improved NUE through GS may reduce the amount of N fertilizer application required in the field, with the environmental benefits that this would bring. Our approach provides a useful tool for plant breeders and researchers. Given the complexity of NUE, screening in controlled environments can eliminate other confounding factors which would be impossible to control in field trials, for example water, temperature, and other mineral nutrients in the soil. Whilst field performance would always be the best method for evaluating lines prior to commercial release, our approach permits the screening of large numbers of candidate lines and selecting those which show most promise before field trials need to commence.

In perennial pasture, some of the nutrients stored in underground organs will mobilize to the leaves following grazing and regrowth. So, to screen perennial pasture plant responses to N supply and NUE, an acclimation period is necessary. One growth cycle of 4 weeks proved to be a suitable time for acclimation in which plant growth response showed no difference between the N treatment levels (**Figure 6**). Results from the two experimental phases were consistent (**Figures 6** and **7**) which indicated good repeatability under these experimental conditions.

### NUE of Perennial Ryegrass

Perennial ryegrass response to N supply has long been of interest to pasture agronomists. Early work showed the response of tiller number, leaf number and leaf area, dry biomass increased along with the increase of N application in field plots (O’Brien, 1960) and sand pot culture (Luxmoore and Millington, 1971). It is as expected that greater biomass production was observed under the moderate N than that under low N (**Table 2**). However, higher N application reduces NUE which is in agreement with previous reports (Jiang et al., 2016). It was found that high plant tissue N content was correlated with low NUE (Garcez and Monteiro, 2016). This may be the primary reason why NUE under moderate N were less than that under low N. Nevertheless, significant variations identified within the breeding population in the present study would be a resource for genomic selection. Since the N concentration of herbage samples were not analyzed in the present study, the differences in NUE attribute to uptake or utilization was unknown.

### Plant Growth

The automated phenotyping greenhouse allows images to be taken within a time series and enables investigation of plant growth response throughout the growth period without sacrificing replicates for destructive harvest (Poiré et al., 2014). It is very useful for outcrossing perennial pastoral grasses under repetitive defoliation. The AGR on MPPA under moderate N supply were almost double that under low N supply. This agrees with the AGR on biomass production under those two N levels. It was reported that greater biomass production under greater N was mainly from increased tiller number and tiller size (weight). The temporal RGR pattern largely resembled the pattern in *Brachypodium* which showed an initial decay phase in the first 3 weeks then became more stable, representing the transition from an early exponential growth stage to a late more linear growth stage (Poiré et al., 2014).

In summary, the NUE and plant growth response to N application were assessed rapidly in a perennial ryegrass breeding population under controlled greenhouse conditions. It provides a tool to screen large populations for genomic selection. The image-based system will be applicable for screening NUE for perennial ryegrass and other comparable forage grasses.

## Data Availability Statement

The raw data supporting the conclusions of this article will be made available by the authors, without undue reservation.

## Author Contributions

JW, PB, and KS designed the experiment. JW and AD conducted the experiment. JW analyzed the data and drafted the manuscript. AD, PB, KS, JW, and GS reviewed and edited the manuscript. GS final approved the submitted version.

## Conflict of Interest

The authors declare that the research was conducted in the absence of any commercial or financial relationships that could be construed as a potential conflict of interest.
